# Transformation of phenolic acids during radical neutralization

**DOI:** 10.1007/s13197-023-05879-w

**Published:** 2023-11-07

**Authors:** Małgorzata Olszowy-Tomczyk, Rafał Typek

**Affiliations:** Department of Chromatography, Institute of Chemical Sciences, Faculty of Chemistry, Maria Curie Sklodowska University, Pl. Marii Curie Sklodowskiej 3, 20-031 Lublin, Poland

**Keywords:** Antioxidant activity, Phenolic acid, Antioxidant transformation, Impact of time, Impact of solvent

## Abstract

Negative influence of reactive oxygen species on living organisms and stability of food products is the reason for significant interest in the substances exhibiting antioxidant properties. Phenolic acids are very popular among the known diet antioxidants. The paper presents the results of research on the application of chromatographic measurements for determination of antioxidant changes and products of radical neutralization reaction for the chosen phenolic acids (caffeic, ferulic, p-coumaric, protocatechuic and vanilic). The measurements were performed using the ABTS method in a long period time (8 days). The experiments have shown that the examined acids were depletion to ca 50 h but the reaction products (dimers or quinones) were converted into new compounds, observed in both methanolic and ethanolic measuring systems after a given period of time. The obtained results seem to be important in the context of living organisms because the biological activity of transformation products and their impact on human health have not been fully recognized yet. Moreover, the presented results can be interesting for both the producer and the consumer of food as generated substances may have influence on the nutritional value as well as the taste and aroma of the food in which phenolic compounds are applied.

**Supplementary Information:**

The online version contains supplementary material available at 10.1007/s13197-023-05879-w.

## Introduction

Generation of reactive oxygen species (ROS) and their negative influence on living organisms and stability of food products are the reason for significant interest in substances exhibiting antioxidant properties, especially of natural origin (Aprioku 2013; Lobo et al. 2010; Sarma et al. 2010).

Of the known exogenous and diet antioxidants one of the largest groups are phenolic compounds (flavonoids, phenolic acids, stilbens, tocopherols, tocotrienols etc.) (Balasundram et al. 2006; Brewer 2011; Foti 2007; Gupta and Sharma 2006) whose main sources are fruits and vegetables. The results of numerous research prove that consumption of phenolic compounds can reduce the risk occurrence of many diseases (Shahidi and Ambigaipalan 2015). In the group of these antioxidants, phenolic acids are particularly common owing to their nutritional value and potential application in many industries (Birošová et al. 2005; Tarnawski et al. 2006).

As results from the literature (Kumar and Goel 2019), the demand for phenolic acids is great in food industry where they are applied for improving the shelf life of perishable products. When added to foods, owing to their antioxidant properties, they are responsible for the control rancidity development, they retard the formation of toxic oxidation products and maintain nutritional quality as well as extend the shelf-life of products. Hence, different kinds of products can be fortificated with phenolic acids because their biological activity can be responsible for protective effects against deterioration of foods as well as for alteration of a given product in functional food (Kumar and Pruthi 2014; Marillanes et al. 2017). Moreover, due to the fact that the phenolic compounds are often perceived by consumers as healthy or natural foods, they can be used for incorporation in the food packaging which is an innovative strategy for the extension of products shelf life. The above-mentioned factors and the fact that the application of phenolic acids in food products leads to a reduction in the use of synthetic substances (providing consumers’ safety when choosing food based on its formulation) cause that the research interest in their properties has been growing (Piazzon et al. 2012; Saxena et al. 2012).

The presented paper is included in the stream of these interests due to the research on the chosen phenolic acids (caffeic, ferulic, p-coumaric, protocatechuic and vanilic acids) and their role as antioxidants. The application of chromatographic measurements for determination of antioxidant changes and products of radical neutralization reaction allowed to find the reply to the question concerning the “fate” of antioxidant and its products of transformation after the process of radical neutralization in both the organism and the food products consumed by it, which is the novelty of the paper.

## Material and methods

### Reagents and equipment

2,2′-Azinobis (3-ethylbenzothiazoline-6-sulfonic acid) diammonium salt (ABTS), potassium persulfate (di-potassium peroxdisulfate), caffeic acid, ferulic acid, p-coumaric acid, protocatechic acid, vanilic acid, acetonitrile, methanol, formic acid, ethanol were purchased from Sigma Aldrich (Poznań, Poland). Water was purified using a Milli-Q system from Millipore (Bedford, MA, USA).

### Measurements of antioxidant changes

#### ABTS method

The measurements of antioxidants changes were performed after the formation of ABTS the cation radical according to Nenadis et al. with some modification (Nenadis et al. 2004; Olszowy-Tomczyk and Typek 2020). For this reason 5 mL of 7 mM aqueous ABTS solution was mixed with 88 μL of 140 mM potassium persulfate (K_2_S_2_O_8_). The mixture was incubated in the dark for 16 h and diluted with methanol (or ethanol) until the absorbance value at 744 nm equalled 2.1. 100 μL of caffeic, ferulic, p-coumaric, protocatechic and vanilic acids in the methanolic or ethanolic solutions (c = 1.5 mg/mL) was mixed with 2000 μL of methanolic or ethanolic solution of ABTS cation radicals. Before each measurement all samples were diluted (1:1 v/v) with water. The chromatographic measurements were performed during the defined reaction time (after 12, 24, 48, 72, 96, 120, 144, 168 and 192 h).

#### HPLC measurements

The chromatographic measurements were performed using a LC/MS system consisting of the UHPLC chromatograph (UltiMate 3000, Dionex, Sunnyvale, CA, USA), a linear trap quadrupole-Orbitrap mass spectrometer (LTQ-Orbitrap Velos from Thermo Fisher Scientific, San Jose, CA) and an ESI source. A Gemini C18 column (4.6 × 100 mm, 3 µm) (Phenomenex, USA) was employed for chromatographic separation performed using the gradient elution. The mobile phase A was 25 mM formic acid in water; the mobile phase B was 25 mM formic acid in acetonitrile. The gradient program started at 5% B increasing to 35% for 100 min, next 35% B to 95% B for 15 min, and ended with the isocratic elution amounting (95% B) for 10 min. The total run time was 125 min at the mobile phase flow rate 0.4 mL/min.

In the course of each run, PDA spectra in the range 190–600 nm and MS spectra in the range 100–2000 *m/z* were collected continuously. In all the antioxidant solutions, the SIM function was used for better visualization of chromatographic separation and removal of the signal regarding the oxidants and reactive radical. In the case of the antioxidants and their derivatives the time periods and monitored ions were as follows:

- Caffeic acid

0.0–22.0 (177 *m/z*), 22.0–22.1 min (178 *m/z*), 22.1–22.5 min (179 *m/z*), 22.5–22.8 min (177 *m/z*), 22.8–23.0 min (178 *m/z*), 23.0–23.6 (179 *m/z*), 23.6–24.7 min (209 *m/z*), 24.7–24.8 min (210 *m/z*), 24.8–27.8 (223 *m/z*), 27.8–100.0 min (224 *m/z*);

- Ferulic acid

0.0–27.9 (193 *m/z*), 27.9–31.0 min (385 *m/z*), 31.0–33.7 min (417 *m/z*), 33.7–34.2 min (385 *m/z*), 34.2–36.6 min (431 *m/z*), 36.6–37.0 (417 *m/z*), 37.0–37.2 min (385 *m/z*), 37.2–38.7 min (417 *m/z*), 38.7–100.0 (431 *m/z*);

- p–Coumaric acid

0.0–27.1 (163 *m/z*), 27.1–33.8 min (325 *m/z*), 33.8–35.3 min (357 *m/z*), 35.3–36.4 min (325 *m/z*), 36.4–39.6 min (357 *m/z*), 39.6–100.0 (371 *m/z*);

- Protocatechuic acid

0.0–17.4 (151 *m/z*), 17.4–17.5 min (153 *m/z*), 17.5–18.5 min (183 *m/z*), 18.5–100.0 min (197 *m/z*);

- Vanilic acid

0.0–22.4 (167 *m/z*), 22.4–100.0 min (333 *m/z*).

For the derivatives of caffeic, ferulic, p-coumaric, protocatechuic and vanilic acids there were used the following MS parameters: the ESI was operated in the negative polarity modes under the following specific conditions: spray voltage—3.5 kV; sheath gas—40 arbitrary units; auxiliary gas—10 arbitrary units; sweep gas—10 arbitrary units; and capillary temperature—320 °C. Nitrogen (> 99.98%) was employed as sheath, auxiliary, and sweep gas. The scan cycle used a full-scan event at the resolution of 60,000. The HRMS analysis was additionally performed for all compounds under examination (see Table 1S in Supporting Information).

**Table 1:** The adducts with MeOH and EtOH observed for the products of examined phenolic acids after 48 h of radical neutralization process

Compounds		MeOH				EtOH			
		tr (min)	Compouds no	*m/z*	c^a^ (μg/mL)	tr (min)	Compouds no	*m/z*	c^a^ (μg/mL)
Caffeic acid	Semiquinone adduct	24.70	1	210	0.048	27.83	10	224	0.059
	Quinone adduct	24.80	2	209	0.390	27.85	11	223	0.500
Protocatechuic acid	Quinone adduct	18.50	3	183	0.368	24.00	12	197	0.654
Ferulic acid	Dimer adduct	33.71	4	417	0.057	36.64	13	431	0.404
		36.97	5		0.015	39.32	14		0.260
		38.70	6		0.010	42.28	15		0.079
p-coumaric acid	Dimer adduct	34.93	7	357	0.005	42.38	16	371	0.015
		35.34	8		0.013	42.82	17		0.034
		39.58	9		0.013	43.49	18		0.018

^a^Amounts were estimated by relating their chromatographic calibration curve for caffeic, protocatechuic and, ferulic, p-coumaric acid

Due to the lack of standards of caffeic, ferulic, p-coumaric, protocatechuic and vanilic acids derivatives, their amounts were estimated by relating their chromatographic calibration curves for the above mentioned acids.

The calibration curve for:

- caffeic acid was used for estimating the amounts of the following caffeic acid derivatives:
  - quinone I, quinone II
  - semiquinone I, semiquinone II
  - quinone adduct with MeOH
  - semiquinone adduct with MeOH
- ferulic acid was used for estimating the amounts of the following ferulic acid derivatives:
  - dimer I, dimer II, dimer III
  - dimer I adduct with MeOH, dimer II adduct with MeOH, dimer III adduct with MeOH
- p-coumaric acid was used for estimating the amounts of the following p-coumaric acid acid derivatives:
  - dimer I, dimer II, dimer III
  - dimer I adduct with MeOH, dimer II adduct with MeOH, dimer III adduct with MeOH
- protocatechuic acid was used for estimating the amounts of the following protocatechuic acid derivatives:
  - quinone
  - quinone adduct with MeOH
- vanilic acid was used for estimating the amounts of the following vanilic acid derivatives:
  - dimer I, dimer II

The chemical structures of phenolic acid derivatives are presented in Fig. 2S (see Supporting Information).

### Statistical analysis

The results are presented as the mean values ± SD. In order to determine the measurements reproducibility, each measurement was repeated three times. The RSD values of all measurements were smaller than 10%. *P* < 0.01 was assumed as statistical difference between the experimental points.

## Results and discussion

**Fig. 1:**
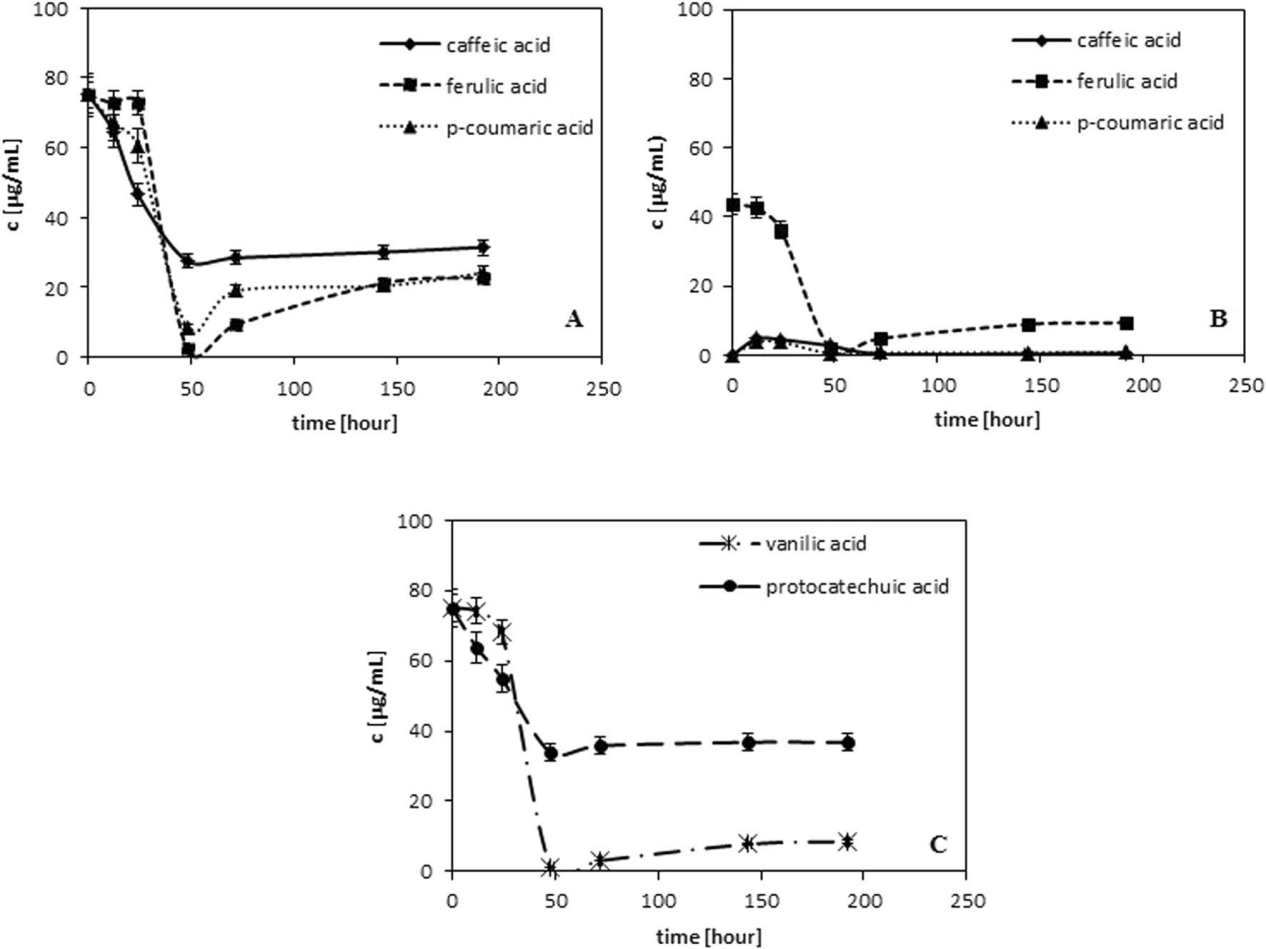
Changes in concentration of chosen antioxidants monitored during 192 h of radical neutralization for: **A** trans caffeic, ferulic and p-coumaric acids. **B** cis caffeic, ferulic and p-coumaric acids. **C** protocatechuic and vanilic acids

Figure 1 presents the changes in the amount of the examined phenolic acids which are monitored during 192 h. In the experiments caffeic, ferulic, p-coumaric, protocatechuic and vanilic acids were used. The choice of these compounds was motivated by the fact that they are commonly found in almost all plants (Shahidi and Wanasundra 2004) and they are applied in food industries (as typical antioxidants, as active components of packaging as well as the components which increase the nutritional and health values in the functional food). As results from literature these naturally occurring phenolic acids can be divided to two groups (Robbins 2003):

- hydroxybenzoic acids with the carboxylic group connected directly with the benzene ring (e. g. protocatechuic and vanilic acids—see Fig. 1S) and,
- hydroxycinnamic acids with the carboxylic group connected by the alkene chain with the aromatic ring (e. g. caffeic, ferulic and p-coumaric acids—see Fig. 1S)

Figure 1A presents the data obtained for caffeic acid, ferulic and p-coumaric acids; Fig. 1B-for their cis forms and Fig. 1C- for vanilic and protocatechuic acids. As results from the obtained data the depletion of antioxidant is observed to ca 50 h of reaction. In all cases at this time the amount of antioxidant decreases then it increases and reaches almost the stable level. This characteristic course of the presented dependencies is observed independently of chemical structures of the antioxidants under investigation.

As results from the literature the phenolic compounds can undergo various changes during radical neutralization (Antolovich et al. 2004; Robbins 2003; Roche et al. 2005). For *ortho*-diphenols AH_2_ (caffeic acid, protocatechuic acid) the radical neutralization proceeds in two steps by formation of semiquinone radicals and quinones during the fast step according to the scheme:

$${\text{AH}}_{{2}} + {\text{ 2R}}^{ \cdot } \to {\text{ A }} + {\text{ 2RH}}$$

**Fig. 2:**
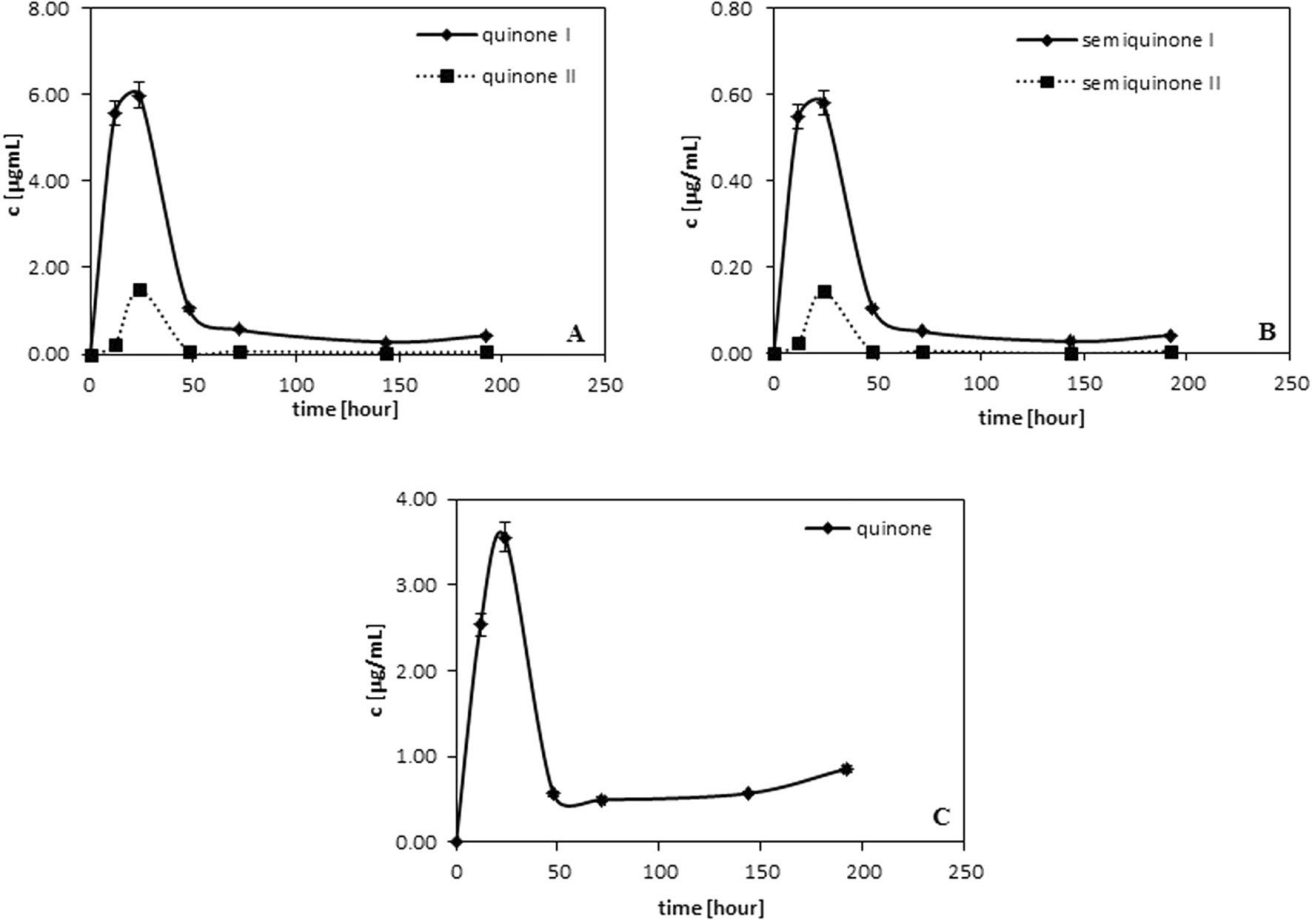
Influence of neutralization time on the concentration of: **A** quinones of caffeic acid. **B** semiquinones of caffeic acid. **C** quinone of protocatechuic acid

As quinone formation is related to the presence of o-hydroxy groups on the benzene ring it is not unusual that in the case of the compounds under investigations, quinones are observed for caffeic and protocatechuic acids. Figure 2 presents the amounts of quinones/or semiquinones in the function of time for caffeic acid (Fig. 2A, B) and for protocatechuic acid (Fig. 2C). As follows from the data in the case of caffeic acid two quinones and two semiquinones are observed. But for protocatechuic acid only one quinone is present in the measurement system. This is associated with the lack of the alkene group of the aromatic ring in the protocatechuic structure (see Fig. 1S). Semiquinones are formed as intermediate products. For protocatechuic acid the semiquinone was not observed which may be due to its quick formation or/and the lack of its stability in the measurement environment. As results from Fig. 2, the largest amounts of quinones (and semiquinones in the case of caffeic acid) are observed between 12 and 24 h of radical neutralization process, then their amounts decrease reaching almost the stable amount after 50 h.

**Fig. 3:**
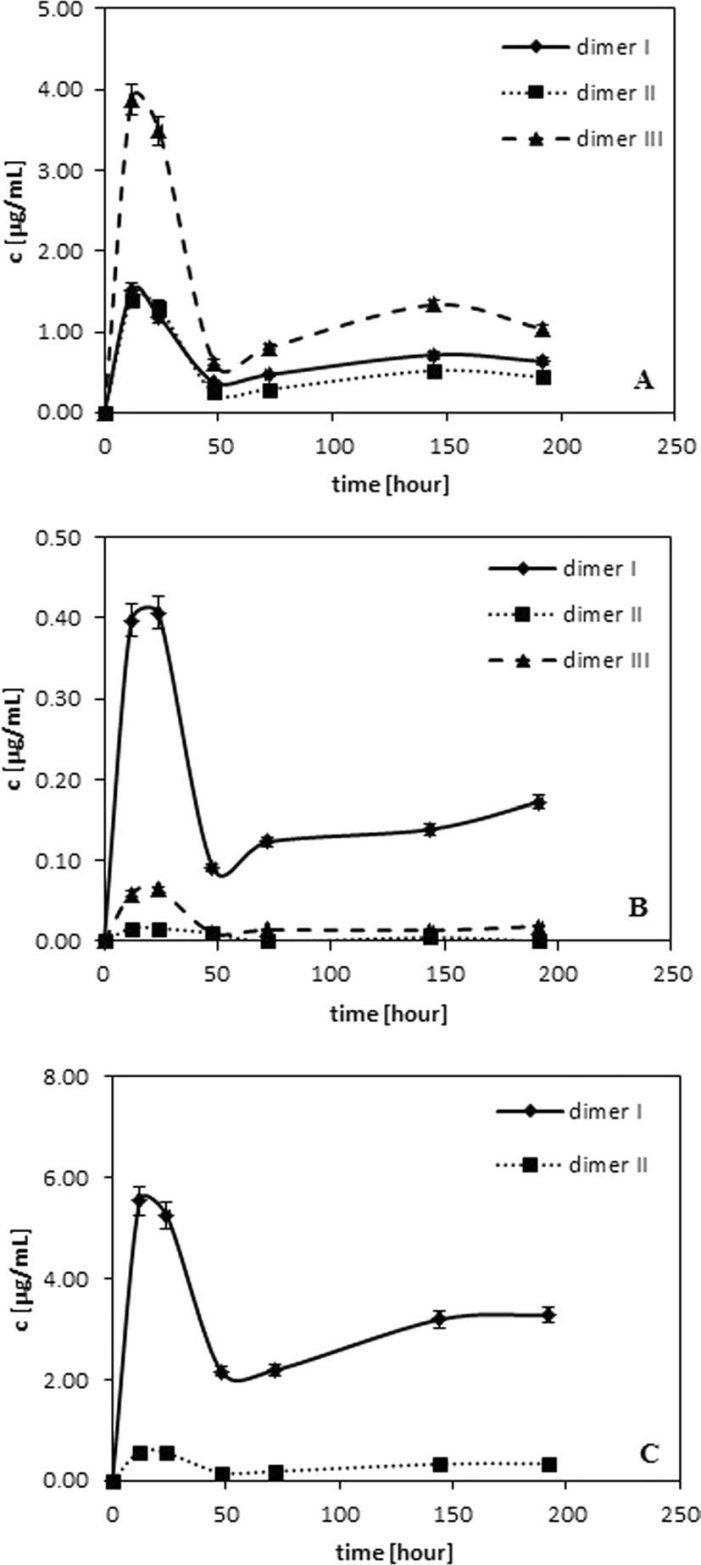
Influence of neutralization time on the amount of: **A** dimers of ferulic acid. **B** dimers of p-coumaric acid. **C** dimers of vanilic acid

Figure 3 shows the amount of dimer in the function of time for ferulic, p-coumaric and vanilic acids. As results from the literature (Roche et al. 2005) monophenols (AH) in the reaction with free radicals must be primarily converted into dimers upon recombination of the corresponding aryloxyl radicals. This reaction occurs according the following scheme:

$${\text{2 AH }} + {\text{ 2R}}^{ \cdot } \to {\text{ A}}_{{2}} + {\text{ 2 RH}}$$

Dimers, similar to quinones, are formed in the largest amounts between 12 and 24 h of radical neutralization, then their amounts decrease and reach almost a constant value. They are generated during phenolic acids depletion (see Fig. 1).

As results from the literature (Dawidowicz and Olszowy 2012) in determination of antioxidant activity a great role is played by solvent which favours dissociation, stabilization of the former product and antioxidant. In the light of the above, it is worth considering the changes of the formed quinones and dimers in different solvents.

The above issue seems to be justified because as follows from Figs. 2 and 3 their amounts are the largest between 12 and 24 h of radical neutralization reaction. Then there are observed some changes in their amounts (decrease).

**Fig. 4:**
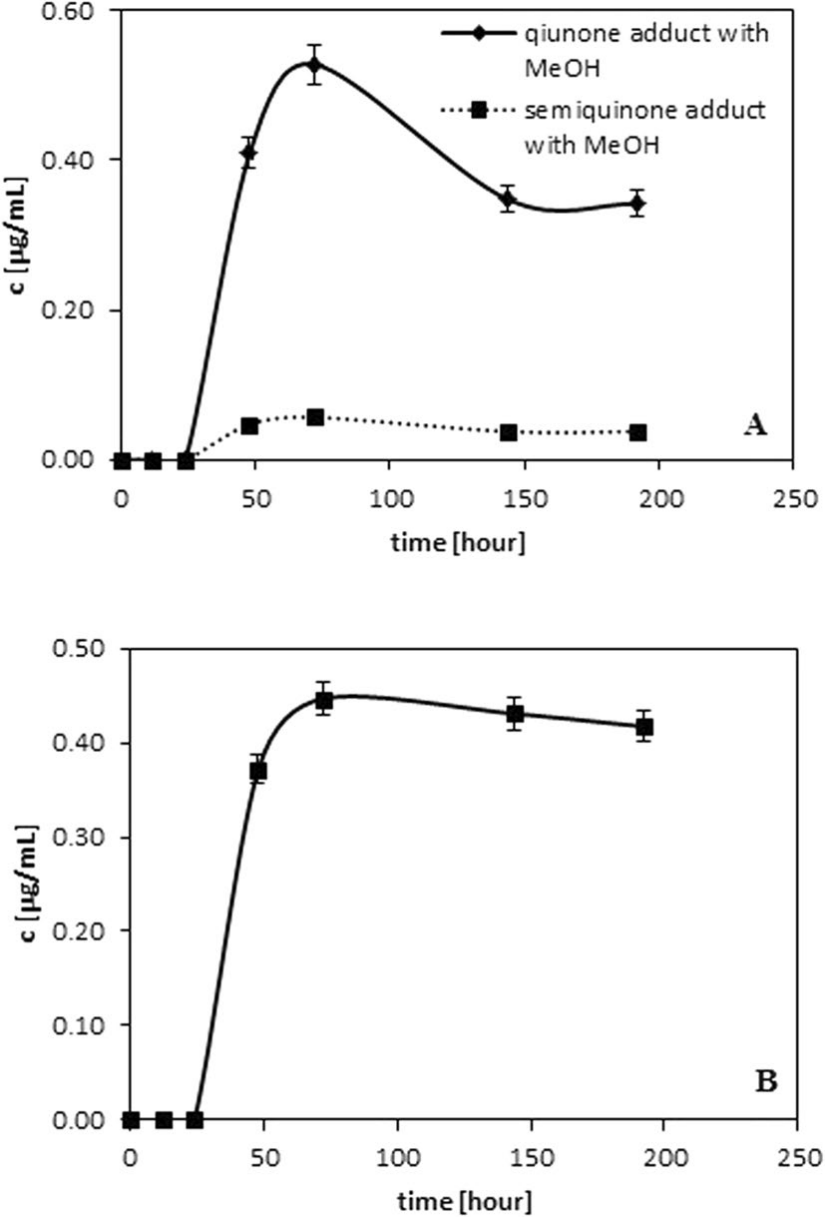
Changes in the amount of quinone/semiquinone adducts with MeOH observed during/after radical neutralization for: **A** caffeic acid. **B** protocatechuic acid

**Fig. 5:**
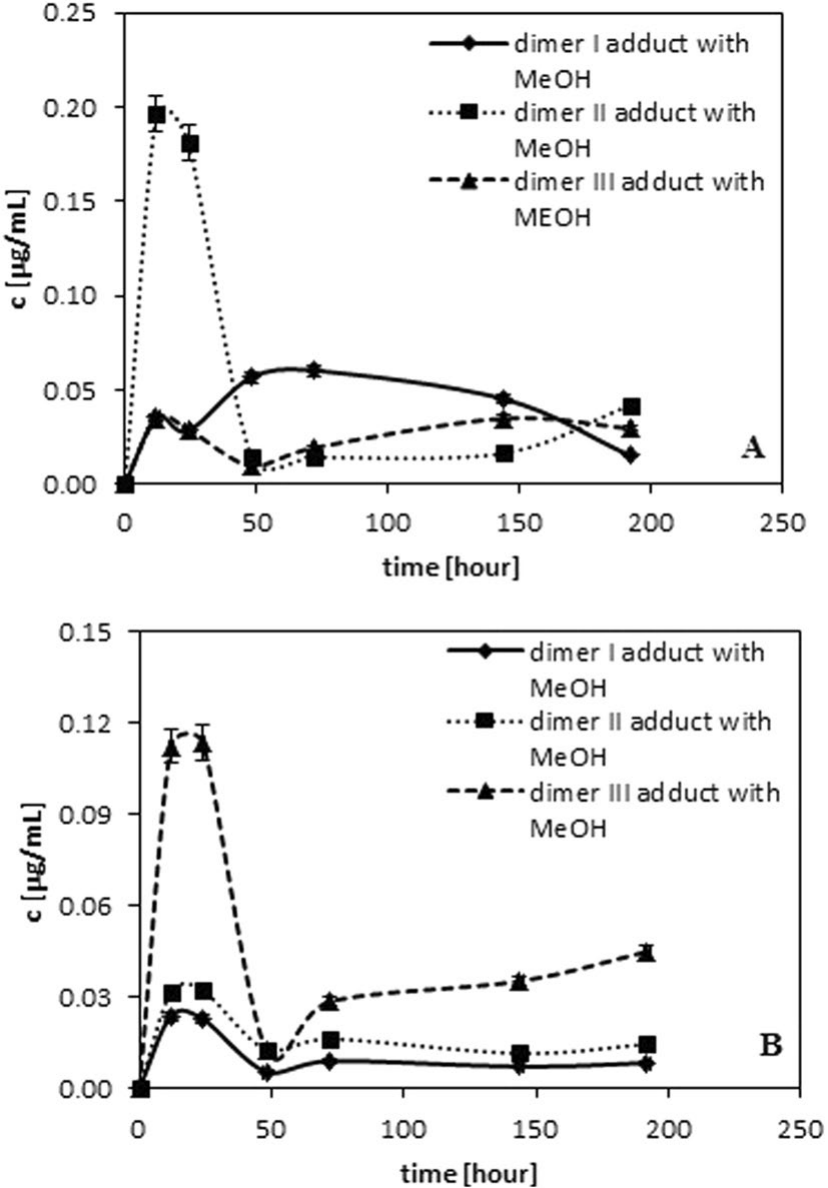
Changes in the amount of dimer adducts with MeOH observed during/after radical neutralization for: **A** ferulic acid. **B** p-coumaric acid

During monitoring the reaction new compounds were found. In terms of *m/z* and longer than parents compounds (quinones, semiquinones and dimers) retention times, they are characterized as the quinones and dimer adducts with methanol (reaction solvent). Figures 4 and 5 show the changes in the amounts of generated adducts in the function of time. As results from the data presented in Fig. 4 quinones and/or semiquinones adducts with MeOH are generated after 24 h of the monitored reaction. In the case of caffeic quinone and caffeic semiquinone (see Fig. 4A), one form of both caffeic quinone adduct and caffeic semiquinone adduct is observed. This can be associated with the fact that the addition of alcohol occurs to the alkene bond. In this way *cis* and *trans* forms disappear. An adduct of the mass about 32 Da higher than that of quinone is observed for the protocatechuic quinone. Figure 1S shows that this phenolic acid belongs to the group of hydroxybenzoic acid and hence the addition of MeOH to protocatechuic acid follows from the reaction of MeOH with the carbonyl group. The literature (Saito et al. 2003) reports that protocatechuic acid is rapidly converted to protocatechuquinone and protocatechuquinone 3-methyl hemiacetal during the reaction with the radical in methanol. Similar behaviour is observed for its another related catechol (Saito and Kawabata 2005). Figure 4 presents the adducts formation after 50 h of reaction which is consistent with the data given in Fig. 2—the decrease of quinones accompanies the increase of their alcohol adducts.

Figure 5 shows the adducts of ferulic dimer (Fig. 5A) and p-coumaric dimer with MeOH (Fig. 5B) formation in the function of time. The alcohol is coordinated to the alkene bonds which can be confirmed by the absence of dimer adduct for vanilic acids (the lack of alkene bond and dimer adducts with methanol). The addition of an alcohol to a double bond, including the –OR group can occur at the α and β positions in relation to the carboxyl group. In the case of this type of compounds, the introduction of a nucleophile in the β position is more probable and privileged- the Michael’s reaction. As results from the data the course of the dependencies is similar to the relationships observed for the data in Fig. 4 (the largest amounts of dimer and dimer adduct are generated between 12 and 24 h of reaction). The experiments revealed that also ethanol is capable of being added to the double bond or/and takes part in formation of hemiacetal.

The information about described adducts (retention time and *m/z*) were collected in Table 1. The numbers in Table 1 correspond to the structure numbers presented in Fig. 2S (see Supporting Information). As it was shown in Table 1, the adducts of dimers and quinones with ethanol are present in the measurement system. Among the methanolic and ethanolic dimers of ferulic and protocatechuic acids, the sequence of the above compounds can be justified as follows:

- working in the RP system, the more polar the compound, the shorter the retention time;
- the more linear the structure, the more easily the analyte enters the column support.

## Conclusion

Phenolic acids are often applied in food industry to improve the shelf life of perishable products, to change a given product in the functional food and to act as an active agent in food packaging. Their usage is associated with their antioxidant properties including their capability of free radical neutralization. The presented research concerns the behavior of chosen phenolic acids during and after neutralization process. The study proves that:

- All examined phenolic compounds take part in reaction during 50 h (at that time their depletion in the measurement system is observed).
- During radical neutralization quinones, semiquinones and dimers are formed (the largest amounts of the listed reaction products are found between 12 and 24 h, then they decrease and reach an almost stable level).
- Adduct quinones, semiquinones and dimers with alcohol are formed during the reaction. The former are generated after 24 h when the amounts of quinones and semiquinones decrease but the amounts of the latter are largest between 12 and 24 h.
- Undoubtedly, in all examined cases the greatest changes for the substrate of reaction and its products are observed between 0 and 72 h. Next the monitored reaction reaches almost a stable state.

As results from the presented experiments phenolic acid oxidation is a really complex process in which many different products can be formed, depending on the structure of examined antioxidant, measurement system components (for example a solvent) and monitoring time.

The quinones and dimers, generated during the radical neutralization, are unstable and may undergo further reaction (often uncontrolled reaction in the living organism). Hence, the obtained results should be important in the context of living organisms because the biological activity of transformation products and their impact on human health have not been fully recognized yet. The presented results can be interesting for both the producer and the consumer of food as generated substances may have influence on the nutritional value as well as the taste and aroma of the food in which phenolic compounds are applied. Moreover, the results, somewhat extended by impact on health, will be helpful in design of functional food whose application will not only provide nutritional benefits but also improves health condition.

### Supplementary Information

Below is the link to the electronic supplementary material.

Supplementary file1 (DOCX 247 kb)

## Data Availability

All data generated or analysed during this study are included in this published article (and its supplementary information files).

## Abbreviations

ABTS: (2,2'-Azino-bis(3-ethylbenzothiazoline-6-sulfonate))
HPLC: High performance liquid chromatography
MS: Mass spectrometry
