# Supplementary material for: Transformation of phenolic acids during radical neutralization
Source: J Food Sci Technol. 2023 Nov 7;61(4):790–7. doi: 10.1007/s13197-023-05879-w (PMC10894153; doi:10.1007/s13197-023-05879-w)

**Table 1S.** HRMS for phenolic acids derivatives

| **Compouds No.** | **Theoretical mass [M–H]¯ (Da)** | **Experimental mass [M–H]¯ (Da)** | **Compounds formula** | **Δ mDa** | **Δ ppm** |
| --- | --- | --- | --- | --- | --- |
| **1** | **209,04500** | **209,04501** | **C10H9O5** | **0,01** | **0,05** |
| **2** | **210,05282** | **210,05271** | **C10H10O5** | **-0,11** | **0,52** |
| **3** | **183,02935** | **183,02922** | **C8H7O5** | **-0,13** | **0,71** |
| **4** | **417,11856** | **417,11872** | **C21H21O9** | **0,16** | **0,38** |
| **5** | **417,11856** | **417,11879** | **C21H21O9** | **0,23** | **0,55** |
| **6** | **417,11856** | **417,11846** | **C21H21O9** | **-0,10** | **0,24** |
| **7** | **357,09743** | **357,09769** | **C19H17O7** | **0,26** | **0,73** |
| **8** | **357,09743** | **357,09717** | **C19H17O7** | **-0,26** | **0,73** |
| **9** | **357,09743** | **357,09771** | **C19H17O7** | **0,28** | **0,78** |
| **10** | **223,06065** | **223,06051** | **C11H11O5** | **-0,14** | **0,63** |
| **11** | **224,06848** | **224,06829** | **C11H12O5** | **-0,19** | **0,85** |
| **12** | **197,04500** | **197,04513** | **C9H9O5** | **0,13** | **0,66** |
| **13** | **431,13421** | **431,13433** | **C22H23O9** | **0,12** | **0,28** |
| **14** | **431,13421** | **431,13403** | **C22H23O9** | **-0,18** | **0,42** |
| **15** | **431,13421** | **431,13439** | **C22H23O9** | **0,18** | **0,42** |
| **16** | **371,11308** | **371,11328** | **C20H19O7** | **0,20** | **0,54** |
| **17** | **371,11308** | **371,11299** | **C20H19O7** | **-0,09** | **0,24** |
| **18** | **371,11308** | **371,11327** | **C20H19O7** | **0,19** | **0,51** |

**Figure 1S.** Chemical structures of the examined antioxidants


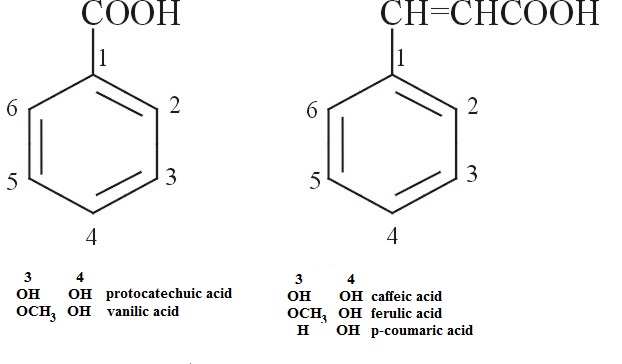


**Figure 2S**. Chemical structures of compounds present in Table1S


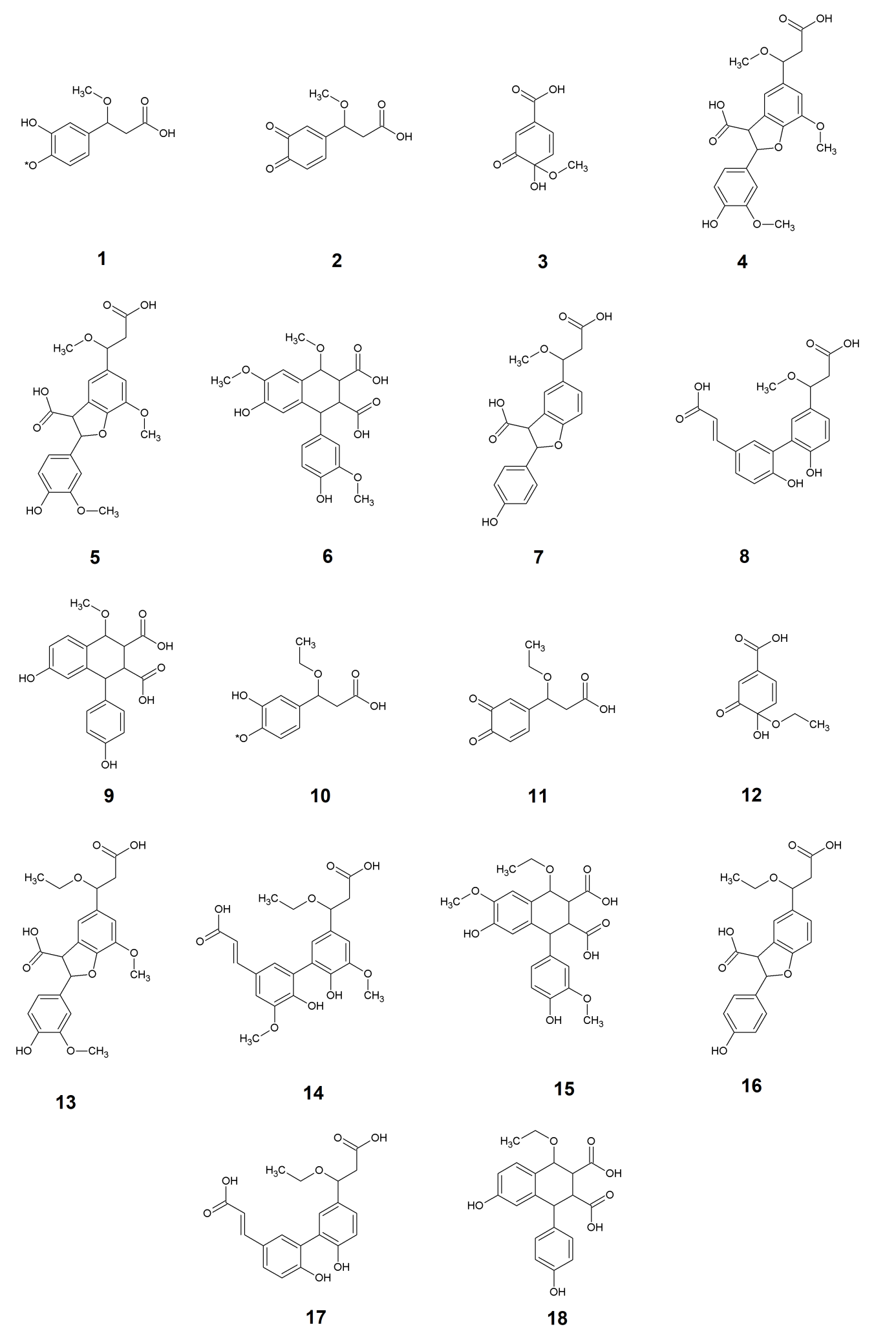

Supplement: Supplementary file 1 — Supplementary file1 (DOCX 247 kb) [file 13197_2023_5879_MOESM1_ESM.docx]
